# Does “Science” Make You Moral? The Effects of Priming Science on Moral Judgments and Behavior

**DOI:** 10.1371/journal.pone.0057989

**Published:** 2013-03-06

**Authors:** Christine Ma-Kellams, Jim Blascovich

**Affiliations:** Department of Psychological and Brain Sciences, University of California Santa Barbara, Santa Barbara, California, United States of America; CSIC-Univ Miguel Hernandez, Spain

## Abstract

**Background:**

Previous work has noted that science stands as an ideological force insofar as the answers it offers to a variety of fundamental questions and concerns; as such, those who pursue scientific inquiry have been shown to be concerned with the moral and social ramifications of their scientific endeavors. No studies to date have directly investigated the links between exposure to science and moral or prosocial behaviors.

**Methodology/Principal Findings:**

Across four studies, both naturalistic measures of science exposure and experimental primes of science led to increased adherence to moral norms and more morally normative behaviors across domains. Study 1 (n = 36) tested the natural correlation between exposure to science and likelihood of enforcing moral norms. Studies 2 (n = 49), 3 (n = 52), and 4 (n = 43) manipulated thoughts about science and examined the causal impact of such thoughts on imagined and actual moral behavior. Across studies, thinking about science had a moralizing effect on a broad array of domains, including interpersonal violations (Studies 1, 2), prosocial intentions (Study 3), and economic exploitation (Study 4).

**Conclusions/Significance:**

These studies demonstrated the morally normative effects of lay notions of science. Thinking about science leads individuals to endorse more stringent moral norms and exhibit more morally normative behavior. These studies are the first of their kind to systematically and empirically test the relationship between science and morality. The present findings speak to this question and elucidate the value-laden outcomes of the notion of science.

## Introduction

Science has stood as a powerful force in shaping human civilization and behavior. As both an ideological system and a method for acquiring information about the world, it offers explanations for the origins of the physical universe and answers to a variety of other fundamental questions and concerns [1]. Past research has noted that personal values influence both the questions that are asked and the methods used in arriving at the answers; as such, scientists have often been concerned with the moral and social ramifications of their scientific endeavors [2], [3]. Not surprisingly, the general consensus is that science is value-laden [4]–[8]. However, no studies to date have directly investigated the link between exposure to science and moral or prosocial behaviors. Here, we empirically examined the effects of thinking about science on moral judgments and behavior.

It is important to note that “science” is multi-faceted construct that takes on distinct forms. On the one hand, the scientific style of thinking employed by scientists is unusual, difficult, and uncommon [9]. Although science can serve as a belief system, it is distinct from other belief systems (e.g., religion) insofar as its counterintuitive nature and the degree to which it does not rely on universal, automatic, unconscious cognitive systems [9]; as a consequence, relative to other belief systems like religion, science has few explicit “followers”. On the other hand, apart from the model of the scientific method of acquiring information about the world, we contend that there is a lay image or notion of “science” that is associated with concepts of rationality, impartiality, fairness, technological progress, and ultimately, the idea that we are to use these rational tools for the mutual benefit of all people in society [10]. Philosophers and historians have noted that scientific inquiry began to flourish when Western society moved from one centered on religious notions of God’s will to one in which the rational mind served as the primary means to understand and improve our existence [10]. As such, the notion of science contains in it the broader moral vision of a society in which rationality is used for the mutual benefit of all.

We predict that this notion of science as part of a broader moral vision of society facilitates moral and prosocial judgments and behaviors. Consistent with the notion that science plays a key role in the moral vision of a society of mutual benefit, scholars have long argued that science’s systematic approach to studying causes and consequences allows for more informed opinions about questions of good and evil [11], and many have argued that the classic scientific ethos stands as an ethically neutral, but morally normative, set of principles that guides scientific inquiry [12]. We contend that the same scientific ethos that serves to guide empirical inquiries also facilitates the enforcement of moral norms more broadly.

## Methods

The ethics committee at the Department of Psychology, University of California, Santa Barbara, specifically approved this study. All participants provided written informed consent.

### Participants

#### Study 1

48 undergraduates (18 men and 30 women ranged 18 to 24 years, mean age = 19.11, *SD* = 1.34) from the University of California, Santa Barbara’s research participation pool were recruited and received course credit for participation.

#### Study 2

33 undergraduates (16 men and 17 women ranged from 18 to 22 years, mean age = 18.67, *SD* = 1.02) from the University of California, Santa Barbara’s research participation pool were recruited and received course credit for participation.

#### Study 3

32 volunteers (16 men and 16 women ranged from 18 to 28, mean age = 20.61, *SD* = 2.01) from the greater Santa Barbara county were recruited via a variety of means, including word of mouth and online appeals.

#### Study 4

43 participants (15 men and 28 women from 18 to 22 years, mean age = 19.35, *SD* = 1.04) from the University of California, Santa Barbara’s research participation pool were recruited and received course credit for participation.

## Design and Procedure

Across four studies, we investigated whether science promotes moral or prosocial behavior. Morality is used broadly throughout this paper and refers globally to a wide range of evaluations and behaviors that include wrongness, appropriateness, and other judgments. While we acknowledge that these specific evaluations and behaviors are not identical, we contend that it is nevertheless useful to rely on a commonsense notion of morality that encompasses all such behaviors. Study 1 used naturalistic measures of exposure to and belief in science and tested whether it predicted the likelihood of enforcing moral norms. Studies 2–4 manipulated thoughts about science and examined the causal impact of such thoughts on both imagined (Studies 2, 3) as well as actual moral behavior (Study 4). Across studies, we examined the effects of science on a broad array of domains, including interpersonal violations (Studies 1, 2), prosocial intentions (Study 3), and economic exploitation (Study 4).

## Materials

### Study 1: Interpersonal Violations

In Study 1, participants read a date rape vignette [13] about John and Sally, two acquaintances who are out on a date. After John drives Sally home, Sally invites him in for a drink; afterwards, John engages in non-consensual sex with her. After reading the vignette, participants were asked judge the wrongness of John’s behavior (i.e., of forcing non-consensual sex with Sally) on a scale from 1 (completely right) to 100 (completely wrong). Afterwards, all participants answered questions regarding their concentrated field of study and the question “How much do you believe in science?” on a scale from 1 (not at all) to 7 (very much). In addition, participants completed basic demographic information (age, sex, whether or not they were religious, ethnicity).

### Studies 2–4: Experimental Manipulations

Studies 2–4 relied on experimental manipulation of science-related vs. control thoughts. Participants were randomly assigned to receive either a science or control prime. The primes involved a series of sentence unscrambling tasks based on the materials used by Shariff and Norenzayan [14]. Both primes involved ten sets of five scrambled words, from which participants had to choose four in order to form a complete sentence. For those in the science condition, half of the sentences contained the key words: logical, hypothesis, laboratory, scientists, and theory. Participants in the control condition completed a similar prime except that all the sentence scrambles contained neutral words (e.g., “shoes give replace old the”; “more paper it once do”).

### Study 2: Interpersonal Violation

In Study 2, all participants read the same vignette about date rape and completed the same moral judgment rating used in Study 1.

### Study 3: Prosocial Intentions

In Study 3, participants completed a prosocial intentions measure [15]. Participants indicated the likelihood of engaging in each of several behaviors in the following month, including prosocial activities (donating to charity, giving blood, volunteering) and distractor activities (attending a party, going on vacation, seeing a movie); the order of activities was randomly presented.

### Study 4: Economic Exploitation

In Study 4, participants completed a behavioral measure of economic exploitation post-experimental manipulation. Participants played an economics dictator game modeled after the procedures used by Shariff and Norenzayan [14]. Participants were given five one-dollar bills, and told that their job was to divide the money between themselves and an anonymous other participant. Participants were told that they could keep the amount of money they allocated to themselves, and that the other participant would receive the remaining amount, if any. Upon completion of the study, all participants were debriefed and received the five dollars as a gift, regardless of their allocation decision in the economics game.

## Results

### Study 1: Interpersonal Violations

Prior to data analysis, participants’ field of study was coded as either a science (e.g. biology, chemistry, physics, psychology) or a non-science field (e.g., art, communication, history, languages/literature, music, sociology, theater).

Gender was not related to any of the variables of interest (all *p*’s >.14), so it will not be discussed. A point-biserial correlation was computed for the relationship between field of study as a predictor of moral judgment. Studying science was positively correlated with both greater moral condemnation of the date rape act (i.e., studying science, relative to studying a non-science field, was associated with rating the act of date rape as more wrong), *r = *.36, *p* = .011. Belief in science in general was also positively correlated with moral condemnation of the date rape act (i.e., those who reported greater belief in science rated the date rape as more wrong), *r* = .65, *p*<.001. Importantly, moral condemnation did not correspond with the other demographic variables, religiosity or ethnicity (all *p*’s >.46).

### Study 2: Interpersonal Violation

There was no main effect of gender nor any gender by condition interactions on the dependent variable of interest in Study 2 (all *p*’s >.22), so gender will no longer be mentioned. In Study 2, those primed with science responded more severely to the moral transgression (i.e., condemned the act as more wrong; *M* = 95.95, *SD* = 4.37) relative to those in the control condition (*M* = 81.57, *SD = *5.09), *F*(1, 31) = 4.58, *p* = .040.

### Study 3: Prosocial Intentions

Likewise in Study 3, there was no main effect of gender nor any gender by condition interactions on the dependent variable of interest. Those primed with science reported greater prosocial intentions (i.e., increased likelihood of donating to charity, giving blood, and volunteering; *M* = 4.14, *SD* = 1.49) relative to those in the control condition (*M* = 3.44, *SD* = 0.98), *F*(1, 31) = 5.64, *p* = .024.

### Study 4: Economic Exploitation

There was a main effect of gender on the dependent variable of interest, money allocated, *F*(1, 55) = 4.98, *p* = .030. Women allocated more money to themselves (*M* = 3.32, *SD = *1.23) than men (*M* = 2.35, *SD* = 1.32). However, no gender by condition interaction emerged, *F*(2, 55) = 1.22, *p* = .30. As predicted, those in the science condition allocated less money to themselves (*M* = 2.71, *SD* = 1.43) than those in the control condition (*M = *2.84, *SD* = 1.11), *t*(41) = 2.06, *p* = .046.

## Discussion

Across the four studies presented here, we demonstrated the morally normative effects of thinking about science. Priming lay notions of science leads individuals to endorse more stringent moral norms (Studies 1, 2), report greater prosocial intentions (Study 3), and exhibit more morally normative behavior (Study 4). The moralizing effects of science were observed both by using naturalistic measures of exposure to science (e.g., field of study) as well as laboratory manipulations of thought-accessibility, and emerged across a broad array of domains, including interpersonal violations (Study 1), academic dishonesty (Studies 2), prosocial behaviors (Study 3), and economic exploitation (Study 4).

It is important to note that the primes used across all studies activated broad, general, lay notions of science rather than specific scientific findings. The key words used the science primes (logical, hypothesis, laboratory, scientists, and theory) were likely associated with semantic notions of rationality, impartiality and progress–notions that are a part of the broader moral view of science as a way of building a mutually beneficial society in which rational tools are used to improve the human condition. The moralizing effects of priming this broad idea of science diverges from previous studies that have focused on the effects of activating specific scientific findings–for example, Vohs and Schooler’s finding that those exposed to scientific findings about humans lacking free will were more likely to cheat [16].

Taken together, the present results provide support for the idea that the study of science itself–independent of the specific conclusions reached by scientific inquiries–holds normative implications and leads to moral outcomes. Previous research has noted that science is value-laden insofar as the extent to which personal values influence both the questions that are asked and the methods used in arriving at the answers [2]–[8]. These findings suggest that beyond these individual differences in previously-formed values that scientists introduce to the process of scientific investigation, the act of thinking about science itself produces important psychological consequences.

The present findings may also help elucidate the effects of subscribing to the broader “ethos” of science. Past scholars have argued that the classic scientific ethos stands as an ethically neutral, but morally normative, set of principles that guides scientific inquiry [12]. These findings suggest the same scientific ethos that serves to guide empirical inquiries also facilitates the enforcement of moral norms more broadly.

Our results should be considered in the light of a number of limitations of our design. First, it is possible that a number of additional factors may have accounted for the natural correlation between exposure to science and enforcement of moral norms in Study 1. Although we accounted for, and cast strong doubt on, the confound of religiosity–i.e., the alternative explanation that greater religiosity predicts both less exposure to science and greater endorsement of moral norms against interpersonal violations–there are nevertheless other factors that may have potentially accounted for the observed relationship. Studies 2–4 serve to address this limitation by relying on experimental primes of science.

Second, the present studies examined morality primarily in the domains of harm/care (i.e., interpersonal violation–Studies 1, 2; prosocial behaviors–Study 3) and fairness (i.e., economic exploitation–Study 4). Existing frameworks regarding the foundations of moral judgments suggest that other moral concerns exist, including authority/respect, ingroup/loyalty, and purity/sanctity [17]. It remains unclear whether science would also exert a moral effect on these additional domains of morality, and the boundary conditions of science’s moralizing outcomes remains an empirical question to be tested in the future.

These limitations notwithstanding, these studies are the first of their kind to systematically and empirically test the relationship between science and morality. No studies to date have directly investigated the link between beliefs in science and moral or prosocial outcomes. The present findings speak to such questions and elucidate the value-laden outcomes of science.
