# Correction: Does “Science” Make You Moral? The Effects of Priming Science on Moral Judgments and Behavior

**DOI:** 10.1371/annotation/be99244d-5b8e-4dca-a3c0-59dbe55c22e8

**Published:** 2013-12-30

**Authors:** Christine Ma-Kellams, Jim Blascovich

The authors would like to make corrections on some of the information reported in the article and provide further clarifications on the analyses carried out, as below:

The N's reported in the main text are correct and the N's reported in the abstract should be updated to reflect those in the main text.

In our studies we never forced participants to answer every question. Thus, some participants choose, either purposefully or inadvertently, to not answer all the questions asked. SPSS, the software we used for data analysis, does listwise deletion of any such cases of missing data.

In Study 2, the values reported after the means are the standard errors of the estimated marginal means, and not the standard deviations of the raw data. This was an inconsistency in reporting, and the SDs of the raw data are 28.02 and 7.69 for the control and experimental conditions, respectively. An examination of the raw data shows that in the control condition, two participants evaluated the date rape act as very low on the immorality scale (scores of 10 and 40 out of 100), which explains the larger SDs in the control condition relative to the experimental. The df reported is the corrected total degrees of freedom rather than the df error.

In Study 3, the denominator for the df's in the F statistic should be 30, and the means should be: M = 2.86, SD = 1.40 for the control vs. M = 4.07, SD = 1.47 for the experimental conditions. The previously reported means were the estimated marginal means when gender is included in the model; however, since gender did not significantly impact the dependent variable of interest, the estimated marginal means should have been replaced with the raw means, indicated above.

In Study 4, the means/SDs should be corrected as follows: M = 3.04 (SD = 1.11) for the control and M = 2.24, SD = 1.43 for the experimental. For the gender effects, the results should read: there was a main effect of gender, F(1, 39) = 4.27, p = .045. Women allocated more money to themselves (M = 2.96, SD = 1.20) than men did (M = 2.27, SD = 1.28); no gender by condition interaction emerged, F(1, 39) = 2.20, p = .14. The previous gender results were the results of an analysis used in the original submission when a third condition was present; however, we dropped the originally included third condition following the recommendation received by a reviewer.

The raw data and syntax for the different studies are available via this Correction.

Science Morality Study 1 Data: http://www.plosone.org/corrections/pone.0057989.001.cn.sav 

Science Morality Study 2 Data: http://www.plosone.org/corrections/pone.0057989.002.cn.sav 

Science Morality Study 3 Data: http://www.plosone.org/corrections/pone.0057989.003.cn.sav 

Science Morality Study 4 Data: http://www.plosone.org/corrections/pone.0057989.004.cn.sav 

Science Morality Study 1 Syntax: http://www.plosone.org/corrections/pone.0057989.005.cn.sps 

Science Morality Study 2 Syntax: http://www.plosone.org/corrections/pone.0057989.006.cn.sps 

Science Morality Study 3 Syntax: http://www.plosone.org/corrections/pone.0057989.007.cn.sps 

Science Morality Study 4 Syntax: http://www.plosone.org/corrections/pone.0057989.008.cn.sps 

