# Correction: Does “Science” Make You Moral? The Effects of Priming Science on Moral Judgments and Behavior

**DOI:** 10.1371/annotation/45c77c00-c715-490d-a21f-58a755f34dcf

**Published:** 2014-01-03

**Authors:** Christine Ma-Kellams, Jim Blascovich

Several errors were included in the published article.

1. The N values listed in the abstract are incorrect. The correct N values should read: study 1 (n = 48), study 2 (n=33), study 3 (n=32), and study 4 (n = 43).

The correct sentences should read Study 1 (n = 48) tested the natural correlation between exposure to science and likelihood of enforcing moral norms. Studies 2 (n = 33), 3 (n = 32), and 4 (n = 43) manipulated thoughts about science and examined the causal impact of such thoughts on imagined and actual moral behavior.

2. In Study 2 in the second sentence of the "Results" section, "SD" should be replaced with "SE". The correct sentence should read: In Study 2, those primed with science responded more severely to the moral transgression (i.e., condemned the act as more wrong; M = 95.95, SE = 4.37) relative to those in the control condition (M = 81.57, SE = 5.09), F(1, 31) = 4.58, p = .040.

3. There is an error in Study 3 where the denominator for the df's in the F statistic should be 30. The correct second sentence of the 'Results' section should read, "Those primed with science reported greater prosocial intentions (i.e., increased likelihood of donating to charity, giving blood, and volunteering; M = 4.07, SD = 1.47 ) relative to those in the control condition (M = 2.86, SD = 1.40 ), F(1, 30) = 5.64, p = .024."

4. The fourth sentence in the 'Results' section should be corrected with the following means: M = 3.04 (SD = 1.11) for the control and M = 2.24, SD = 1.43 for the experimental. The corrected sentence should read: As predicted, those in the science condition allocated less money to themselves (M = 2.24, SD = 1.43) than those in the control condition (M = 3.04 SD = 1.11), t(41) = 2.06, p = .046.

5. The first three sentences of the results section of Study 4 should be replaced with the following text: There was a main effect of gender, F(1, 39) = 4.27, p = .045. Women allocated more money to themselves (M = 2.96, SD = 1.20) than men did (M = 2.27, SD = 1.28); no gender by condition interaction emerged, F(1, 39) = 2.20, p = .14. 

